# Dental pain, oral impacts and perceived need for dental treatment in Tanzanian school students: a cross-sectional study

**DOI:** 10.1186/1477-7525-7-73

**Published:** 2009-07-30

**Authors:** Kijakazi O Mashoto, Anne N Åstrøm, Jamil David, Joyce R Masalu

**Affiliations:** 1 Department of Clinical Odontology, University of Bergen, Bergen, Norway; 2 Centre for International Health, University of Bergen, Bergen, Norway; 3 National Institute for Medical Research, Dar es Salaam, Tanzania; 4 Faculty of Dentistry, Muhimbili University of Health and Allied Sciences, Dar es Salaam, Tanzania

## Abstract

**Background:**

Dental caries, dental pain and reported oral problems influence people's oral quality of life and thus their perceived need for dental care. So far there is scant information as to the psychosocial impacts of dental diseases and the perceived treatment need in child populations of sub-Saharan Africa.

**Objectives:**

Focusing on primary school students in Kilwa, Tanzania, a district deprived of dental services and with low fluoride concentration in drinking water, this study aimed to assess the prevalence of dental pain and oral impacts on daily performances (OIDP), and to describe the distribution of OIDP by socio-demographics, dental caries, dental pain and reported oral problems. The relationship of perceived need estimates with OIDP was also investigated.

**Methods:**

A cross-sectional study was conducted in 2008. A total of 1745 students (mean age 13.8 yr, sd = 1.67) completed an extensive personal interview and under-went clinical examination. The impacts on daily performances were assessed using a Kiswahili version of the Child-OIDP instrument and caries experience was recorded using WHO (1997) criteria.

**Results:**

A total of 36.2% (41.3% urban and 31.4% rural, p < 0.001) reported at least one OIDP. The prevalence of dental caries was 17.4%, dental pain 36.4%, oral problems 54.1% and perceived need for dental treatment 46.8% in urban students. Corresponding estimates in rural students were 20.8%, 24.4%, 43.3% and 43.8%. Adjusted OR for reporting oral impacts if having dental pain ranged from 2.5 (95% CI 1.8–3.6) (problem smiling) to 4.7 (95% CI 3.4–6.5) (problem sleeping),- if having oral problems, from 1.9 (95% CI 1.3–2.6) (problem sleeping) to 3.8 (95% CI 2.7–5.2) (problem eating) and if having dental caries from 1.5 (95% CI 1.1–2.0) (problem eating) to 2.2 (95% CI 1.5–2.9) (problem sleeping). Students who perceived need for dental care were less likely to be females (OR = 0.8, 95% CI 0.6–0.9) and more likely to have impacts on eating (OR = 1.9, 95% CI 1.4–2.7) and tooth cleaning (OR = 1.6, 95% CI 1.6–2.5).

**Conclusion:**

Substantial proportions of students suffered from untreated dental caries, oral impacts on daily performances and perceived need for dental care. Dental pain and reported oral problems varied systematically with OIDP across the eight impacts considered. Eating and tooth cleaning problems discriminated between subjects who perceived need for dental treatment and those who did not.

## Background

The usefulness of oral health related quality of life, OHRQoL assessments depends on their ability to predict important outcomes and to detect intervention related change [1]. Few attempts have been made to evaluate OHRQoL, and to describe its relationship with perceived dental treatment need in child-and adolescent populations of developing countries [2,3]. This is notable since children represent a major focus of dental public health care globally. Moreover, paediatric oral disorders are numerous and likely to affect children's OHRQoL negatively [4,5]. Instruments are now available for measuring OHRQoL in school-aged children, such as the Child Perceptions Questionnaire [4] and the Child Oral Impacts on Daily Performance (OIDP) inventory [5]. The Child-OIDP was developed and tested among Thai schoolchildren aged 11–12 yr [5]. It has been found to be a reliable and valid instrument when applied to children in numerous countries, such as Thailand, France, UK and Tanzania [5-8].

Untreated dental caries might lead to dental pain and impact daily activities in terms of play, sleep, eating and school activity [9]. In Tanzania, the exposure to dental services is low particularly in the rural areas and although dental caries prevalence has remained low in the child population, dental pain and discomfort have been cited as common reasons for seeking dental care [10]. The primary model of treatment is tooth extraction with negligible contribution from restorative care [10-12]. Information on the extent, distribution and psycho-social impacts of dental pain is important when assessing children's burden of oral diseases and their perceived need for dental care [3]. Reportedly, the main benefits of dental treatment relate to improved psychological and social well-being [13]. Thus, oral symptoms and impacts on daily activities might constitute an adjunct assessment of perceived dental treatment need [13,14]. To date, dental pain and its psychosocial consequences pertaining to the child populations of Sub-Saharan Africa has been given little attention in the literature and the relationship of oral impacts with perceived dental treatment need has yet to be investigated.

In a review of the literature considering dental pain among children and adolescents, Slade [9] reported the prevalence of toothache to range from 5% to 33% across various countries. Shepherd et al. [15] interviewed 8-yr-old British children and found a prevalence of dental pain of 47.5%. In non-industrialized countries, the prevalence and severity of children's dental pain has usually been higher than the figures presented from UK, the USA and Europe [16,17]. In a study of Ugandan secondary school children, aged 13–19, toothache in the last four weeks was estimated to 36.5% [18]. Focusing on Ugandan primary schoolchildren, 10–14 yrs, Kiwanuka and Åstrøm [19] reported on a prevalence of dental pain during the last 12 months amounting to 42% and 52% in boys and girls, respectively. Recently, the reported prevalence of toothache during the previous 12 months was estimated to 41% in 11-, 13- and 15-yr- old Chinese schoolchildren [20] and to 30% in 11–14-yr- old Pakistani schoolchildren [3]. Dental pain has been reported to be prevalent among children even in contemporary populations with historically low levels of caries experience [9]. In the health and lifestyle survey conducted among Finnish adolescents, 1977–1997, no tendency for the prevalence of toothache to decline across time was recorded despite a corresponding decline in caries experience [21]. Nevertheless, caries – toothache associations are found to be strongest in populations with reduced access to dental care, in lower socio-economic status groups and in populations where dental caries is largely untreated [9].

### Purpose

Focusing on primary schoolchildren resident in Kilwa, south-eastern Tanzania, this study aimed to assess the prevalence of dental pain and oral impacts on daily performances (OIDP), and to describe the distribution of OIDP by socio-demographics, dental caries, dental pain and reported oral problems. The relationship of OIDP with perceived dental treatment need was investigated in an attempt to assess the predictive validity of the Child-OIDP frequency questionnaire in the context of primary schoolchildren in rural Tanzania.

## Methods

### Study area

The present paper is based on data generated from a cross-sectional baseline study, which is part of a prospective intervention that was implemented in Lindi region from April to September 2008. Lindi, a coastal region located in south-eastern Tanzania, is one of the most sparsely populated regions of Tanzania main land with a population density of 66,046 per square km. The population was 791,306 as of the 2002 national census [22]. Lindi region is divided into six districts; Lindi urban (N = 41,549), Lindi rural (N = 215,764), Liwale (N = 75,546), Ruangwa (N = 124,516), Nachingwea (N = 162,081) and Kilwa (N = 171,850). Kilwa district was purposively selected for this study, since the fluoride concentration in water (0.2 mg/L) is low and since the district is particularly deprived with respect to oral health care services. The district of Kilwa is bordered in the north by the area of Coastal region, in the east by the Indian Ocean, in the south by the rural district of Lindi and in the west by the district of Liwale.

### Study population

Kilwa district is divided into 20 wards, of which 18 are rural (N = 7444) and 2 are urban (N = 1165). The study population comprised of 10–19 yr-olds attending standard 6 in public primary schools in Kilwa district. As this study included several outcomes, the size of the sample was calculated separately for each of them and the largest sample size required was adopted. A sample size of 2000 primary school children was calculated to be satisfactory; assuming that the percentage of children expected to have dental caries was 30%, using an absolute precision (d) of 0.03, 95% CI and a design factor of 2 [23]. Some of the schools in the selected wards were not accessible due to natural calamities in the area at the time of data collection. Moreover, the number of enrolled subjects and attendance rates in rural schools were particularly low. To reach the estimated sample size, 8 rural wards (8/18 = 0.4) were selected at the first stage by systematic random sampling. In addition both urban wards were included in the sample. At the second stage, standard 6 pupils in all primary public schools that were accessible in the urban and in the 8 selected rural wards were included in the sample. A total of 27 schools (N = 2465, 17 rural n = 1408 and 10 urban n = 1059) out of a total of 101 schools (N = 8609, urban = 1165 and rural = 7444) present in Kilwa district were invited to participate in the study (n = 2467). The official age for entry into the primary level is 7 yrs and the official primary level of schooling is seven standards (grade 7). Thus, grade 6 pupils were expected to be 12 – 15-yrs- old. Permission for participation was sought from school authorities and from parents when pupils were below 18 yrs. Ministry of Education and Vocational Training through the District Council approved the conduct of the study. Ethical clearance was granted by the National Institute for Medical Research in Tanzania and the Regional Committee for Medical Research Ethics and the Norwegian Data Inspectorate. Written and verbal informed consent to participate in the study was obtained from schoolchildren and their parents.

### Interview

A structured interview schedule, covering socio-demographics and various aspects of oral health was administered by trained and calibrated research assistants and completed by the pupils in face to face interviews. The questionnaire was originally constructed in English, translated to Kiswahili, the national language of Tanzania, and then back translated into English. The questionnaire was pilot tested prior to its use in the field. Each interview was conducted in a private and quiet place outside the classroom. *Oral health related quality of life *was measured using a Kiswahili version [8] of the eight item Child OIDP inventory (e.g. During the previous 3 months – how often have problems with your teeth and mouth caused you any difficulty with; eating, speaking, cleaning teeth, smiling, sleeping, emotional balance, study and social contact). The students completed the Child-OIDP frequency questionnaire at school in face to face interviews administered by two trained research assistants before the clinical examination. The interview started with the students reviewing common oral problems, in terms of "toothache, sensitive teeth, problems with position of teeth, ulcer in mouth, bleeding in mouth, swollen gums, bad breath, problems with color of teeth, problems with spaces of teeth, other problems" and options given were (1) yes or (2) no whether they had experienced them during the previous 3 months. The Child-OIDP frequency index referred to difficulty carrying out eight daily life activities, each scored 0–3 where (0) never, (1) once or twice a month, (2) once or twice a week, (3) every day/nearly every day [8]. The total Child-OIDP score was constructed in two ways. First, by adding the 8 performance scores as originally scored (0–3) into a Child-OIDP additive score (ADD) (range 0–24). Second, the Child-OIDP simple count (SC) score (range 0–8) was constructed by summing the dichotomized frequency items of (1) affected and (0) not affected. The Kiswahili version of the OIDP frequency questionnaire has previously been tested for validity and reliability in population-based studies involving urban primary school children in Dar es Salaam [8]. *Dental pain *was computed by combining toothache and tooth sensitivity into a sum score with the categories (0) no dental pain and (1) dental pain reported. A sum score of *reported oral problems *was computed from questions on broken tooth, position of teeth, swollen gums, bad breaths, and ulcers in the mouth, bleeding gums, colour of the teeth and gum abscess. This score was dichotomised into (0) no reported oral problems, (1) reported at least one oral problem. *Self assessed oral health *was assessed asking: "What do you think about the state of your teeth and mouth?" The responses ranged from (1) very good to (4) very bad. "How satisfied or dissatisfied are you with your teeth or mouth, tooth appearance, tooth colour, position of teeth, and chewing ability"? The responses for the five questions ranged from (1) very satisfied to (4) very dissatisfied. A sum score for self-rated oral health was obtained by adding the six items and then dichotomised into (0) good/satisfied and (1) poor/dissatisfied. *Perceived dental treatment need *was measured by the response to the question "Do you perceive any need for dental treatment at the moment? The response was either yes (1) or no (0). *Parents' level of education *was originally scored from (1) no education to (6) college or university education. For analysis the variables (mother's and father's education) were recoded into (0) low education (including original categories 1 and 2) and (1) high education (including original categories 3, 4, 5 and 6). *Family wealth *was assessed as an indicator of socio-economic status according to a standard approach in equity analysis [24]. Durable household assets indicative of family wealth (i.e. bicycle, motorcycle, car, TV) were recorded as (1) "available and in working condition" or (0) "not available and/or not in working condition." These assets were analyzed using principal components analysis, PCA. The first component resulting from this analysis was used to categorize households into four approximate quartiles of wealth ranging from the 1^st ^poorest quartile to the least poor 4^th ^quartile

### Clinical examination

Clinical examination was carried out by one trained and calibrated dentist (KOM). Cotton roles were used to control saliva. Caries experience was assessed under field conditions using natural light, probes and mouth mirror according to the criteria described by the World Health Organization, WHO [25]. DMFT was computed as the sum of decayed, missed and filled teeth. Examined students were categorized into those who were caries free DMFT = 0 and those with caries experience DMFT>0. Lesions were recorded as present when a carious cavity was apparent on visual inspection. A tooth was considered missing if there was a history of extraction because of pain and/or a cavity prior to extraction

### Test-retest reliability

Duplicate clinical examinations were carried out on a randomly selected sub-sample of 20 participants in one school. Kappa values for both decayed permanent teeth and DMFT intra-examiner agreement was 1. Kappa values for missing and filled teeth could not be computed as the sub-sample selected had no missed and filled teeth

### Statistical analysis

Data were analysed using the Statistical Package for Social Science (Version 15.0.1). Cluster effect was adjusted for using STATA 10.0. Cross tabulations were tested by Chi-square statistics. Internal consistency reliability was assessed using Cronbach's alpha. Construct validity was determined by comparing OIDP scores of groups that differ regarding self reported oral health status. Multivariate analyses with OIDP and perceived dental treatment need as outcome variables were conducted using multiple logistic regression analyses and 95% Confidence intervals (CI). A forced entry method was used during logistic regression analyses and the level of significant was set at 0.05

## Results

### Sample profile

A total of 1780 (1780/2465, response rate 72.6%) with mean age of 13.8 yrs (standard deviation (sd) 1.67) consented to participate in the study. Being out of school at the time of data collection was the main reason for non-participation. Twelve students below the age of 10- and above the age of 19 yr were excluded from the analysis. Moreover, 23 subjects refused to be examined clinically because of fear of the dental instruments. A total of 837 students from urban (52.3% girls, mean age 13.4 [sd 1.62]) and 908 from rural; 48.5% girls, mean age 14.2 [sd 1.64]) completed an extensive personal interview and under-went a full mouth clinical examination. As shown in Table 1, socio-demographic variables and self-reported oral health varied systematically with urban-rural place of residence. Urban residents reported dental pain and other oral problems more frequently than rural residents. Urban participants had parents with higher education and belonged to the least poor 4^th ^quartile on the family wealth index more frequently than did rural participants.

**Table 1:** Socio-demographic characteristics of participants according to place of residence

	Urban % (n)	Rural % (n)	All % (n)
Age:			
10 – 14 years	77.3 (647)	59.1 (537)	67.9 (1184)
15 – 19 years	22.7 (190)	40.9 (371)**	32.1 (561)
Sex:			
Male	47.7 (399)	51.5 (468)	49.7 (867)
Female	52.3 (438)	48.5 (440)	50.3 (878)
Mother's education:			
Low	38.3 (325)	50.1 (455)	44.7 (780)
High	61.2 (512)	49.9 (453)**	55.3 (965)
Father's education:			
Low	39.2 (328)	43.8 (398)	41.6 (726)
High	60.8 (509)	56.2 (510)*	58.4 (1019)
Family wealth index:			
1^st ^quartile (Poorest)	22.2 (186)	30.3 (275)	26.4 (461)
2^nd ^quartile	33.6 (281)	55.0 (499)	44.7 (780)
3^rd ^quartile	5.7 (48)	2.5 (21)	4.0 (69)
4^th ^quartile (Least poor)	38.5 (322)	12.4 (113)**	24.9 (435)
DMFT>0	17.4 (146)	20.8 (189)	19.2 (335)
Dental pain:			
Yes	36.4 (305)	24.4 (222)*	30.2 (527)
Reported dental problems:			
Yes	54.1 (453)	43.3 (393)*	48.5 (846)
Perceived need for dental care:			
Yes	46.8 (392)	43.8 (398)	45.3 (790)

**p < 0.001; *p < 0.05

### Reliability and construct validity of the Child OIDP questionnaire

In the present study, all participants completed the Child-OIDP frequency inventory, providing support to its face validity. Internal consistency reliability (standardized item alpha) was 0.85 and 0.84 among urban and rural residents, respectively. The inter item correlations ranged from 0.29 (contact people) to 0.51 (speaking/sleep and smile/emotion). The corrected item total correlation (i.e. the correlation between each item and the total score omitted for that item) ranged from 0. 54 (eating) to 0.63 (sleeping), being above the minimum level of 0.20 for including an item into scale [26] Construct validity was demonstrated in that Child-OIDP scores increased as the students' self-reports of oral health changed from healthy to unhealthy. Thus, a total of 27.9% versus 82% (p < 0.001) of the participants reporting good and bad dental condition had experienced at least one OIDP.

### Prevalence of dental caries, self reported pain and self reported oral problems

The mean DMFT scores were 0.37 (sd 0.85) and 0.32 (sd 0.79) in urban and rural students, respectively. The crude and age standardized (in parenthesis) estimates of DMFT>0 were 17.4% (19.1%), dental pain 36.4% (36.7%), other oral problems 54.1% (54.1%) and perceived treatment need 46.8% (46.8%) in urban students. Corresponding estimates in rural students were 20.8% (20.9%), 24.4% (24.5%), 43.3% (43.3%) and 43.8% (48.1%) (Table 1). Of students with DMFT>0, 51.3% and 54.0% confirmed dental pain and other oral problems, respectively (not shown in the table).

### Prevalence and correlates of OIDP

A total of 36.2% (crude prevalence rate; 41.3% urban, 31.4% rural, p < 0.001, age standardized prevalence rate; 41.5% urban and 31.4% rural) reported at least one OIDP. The most and least frequently reported oral impact in urban students were eating (22.8%) and smiling problems (12.5%). Corresponding figures in rural students were cleaning (16.4%) and school work-, smiling-, emotion- and speaking problems (10.2% to 10.5%) (not in table). In the urban area, among subjects with impacts, 29.7%, 20.3% and 6.0% had respectively, 1, 2 and 8 oral impacts. Corresponding figures among rural residents were 27.6%, 25.6% and 7.2%. Place of residence varied systematically with OIDP across all impacts, except problems smiling and social contact with urban students reporting each impact more frequently than their rural counterparts (See table S1; additional file 1). Students in the 3^rd ^quartile of the family wealth index reported problems eating and problems cleaning more frequently than those in the 1^st ^quartile. Dental caries experience, reported pain and oral problems varied systematically with OIDP across the eight impacts investigated. Caries free students, those reporting no pain and those who had no oral problems, experienced oral impacts less frequently than their counterparts in the opposite groups. The least poor students, according to the family wealth index, reported dental pain and other oral problems more frequently than their counterparts in the poorest 1^st ^and 2^nd ^quartiles (not shown in table).

To adjust for potential confounding factors, the association of each OIDP item with dental caries, dental pain and reported oral problems were estimated in multiple logistic regression analyses, adjusting for place of residence, gender, age, family wealth index and parental education. The adjusted ORs for experiencing oral impacts if having dental caries were 1.5 (95% CI 1.1–2.0) regarding problems eating, 2.2 (95% CI 1.5–2.9) regarding problems sleeping and 1.5 (95% 1.0–2.0) regarding problems with school work. Adjusted OR's for having impacts if reporting pain and experiencing other oral problems are depicted in table S2; additional file 2. Model fit in terms of Nagelkerke's R^2 ^ranged from 0.114 (11.4%) difficulty smiling to 0.259 (25.9%) difficulty eating.

### Predictive validity of OIDP

Using multiple logistic regression with perceived need for dental treatment as outcome variable, all OIDP items and family wealth index were entered simultaneously whilst controlling for age, gender, place of residence and parental education. Those perceiving need for dental treatment were more likely to have problems eating (OR = 1.9, 95% 1.4 – 2.7) and cleaning (OR = 1.6, 95% CI 1.2 – 2.5) compared to their counterparts without perceived need for dental treatment. Girls were less likely to perceive need than boys (OR = 0.8, 95% CI 0.6 – 0.9) (Table 2). Once the main effects were established, all pairwise interaction effects were examined. Two-way interactions occurred between problems sleeping and problems eating on the one hand side and urban/rural residence on the other. Stratified analyses with urban and rural participants revealed that whereas eating and tooth cleaning problems were the most important predictors of perceived need in urban schoolchildren, eating problems and sleeping problems were the strongest predictors in their rural counterparts (Table 2). Both problem eating and problem sleeping presented a statistically significantly stronger relationship with perceived treatment need in rural- than in urban schoolchildren.

**Table 2:** Perceived need for dental care regressed on socio-demographics and OIDP items- adjusted for age, gender, place of residence and parental education

	Unadjusted % (n)	Adjusted total OR (95% CI)	Adjusted urban OR (95% CI)	Adjusted rural OR (95% CI)
Male	48.0 (416)	1	1	1
Female	42.6 (374)	0.8 (0.6–0.9)	0.7 (0.5–1.0)	0.8 (0.6–1.1)
^a^1^st ^quartile (poorest)	42.8 (199)	1	1	1
2^nd ^quartile	42.8 (1991)	1.0 (0.8–1.2)	0.8 (0.5–1.2)	1.0 (0.7–1.4)
3^rd ^quartile	58.0 (40)	1.6 (0.9–2.8)	1.5 (0.7–2.9)	1.5 (0.5–4.2)
4^th ^quartile (least poor)	49.5 (219)*	1.2 (0.9–1.6)	1.0 (0.7–1.5)	1.2 (0.7–4.9)
Eat problem				
No	40.0 (563)	1	1	1
Yes	67.6 (227)**	1.9 (1.4–2.7)	1.4 (1.0–2.1)	2.9 (1.7–4.9)
Cleaning				
No	40.3 (566)	1	1	1
Yes	65.9 (224)**	1.6 (1.2–2.5)	2.0 (1.3–3.0)	1.2 (0.7–2.0)
Sleep				
No	8.9 (85)	1	1	1
Yes	21.4 (169)**	1.2 (0.8–1.8)	1.0 (0.6–1.7)	1.8 (1.0–3.1)
Nagelkerke's R^2^	0.093	0.089	0.076	0.14

*p < 0.05, **p < 0.0001 ^a ^family wealth index

To explore the social dependency of perceived need further, the two indicators of OIDP and perceived treatment need were cross-tabulated. Among urban schoolchildren, a total of 49.7% (59% of discordant pairs) perceived treatment need in spite of having no oral impacts. Corresponding figures in the rural areas was 52.3%. In urban, 33.8% (44% of discordant pairs) reported no treatment need whilst they nonetheless reported oral impacts. Corresponding figure in rural area was 18.5% (31% of discordant pairs). The latter discrepancy amounted to 13%, 11.7%, 18.8% and 18.6% (p < 0.001) of children belonging to the 1^st^, 2^nd^, 3^rd ^and 4^th ^family wealth index category, respectively.

## Discussion

This article reported upon the prevalence of dental caries experience, dental pain, other oral problems and oral impacts on daily performances in a deprived population of 10–19-yr- olds attending primary school in Kilwa district, Tanzania, detailed the association of clinical- and self-reported oral health indicators with OIDP and examined which oral impacts on daily activities affected perceived dental treatment needs. In spite of a low prevalence of untreated dental caries (19.2%), dental pain, oral problems and oral impacts affected a significant part of the population studied. Moreover, whereas dental caries and reported oral problems were useful predictors of OIDP, OIDP, in turn predicted perceived dental treatment needs accounting for between 8% and 14% of its explainable variance. In presenting unweighted prevalence estimates, the present study is limited in that the sample was not self-weighted and thus differed in some aspects from the population of urban/rural schoolchildren considered. This should be taken into consideration when interpreting the findings pertaining to the urban and rural schoolchildren combined.

According to the present data, the 3 months period prevalence of dental pain (including tooth sensitivity) and reported oral problems of Kilwa students amounted to 30% and 48.5%, respectively. The corresponding prevalence rates in students with caries experience were 50% and 54%, respectively. Obviously, if toothache and tooth sensitivity had been assessed separately, the prevalence estimates of dental pain would have differed. Nevertheless, the present results are within the range of dental pain prevalence rates reported by Slade [9] and accord with the 1-month period prevalence of dental pain observed among similar aged children and adolescents in Uganda, Pakistan, China, Greece, UK and Brazil [18-20,27-29]. Comparing the present prevalence rates across young populations worldwide should be done with caution since various time frames and age groups are focused in the different studies. Using a relatively long recall period of 3 months might have led to a slight underestimation of the prevalence rates reported in this study. Evidently, however, experience from Tanzania have indicated that a recall period for up to 12 months does not affect the prevalence estimates when it comes to more serious experiences (e.g. toothache) [30]. The causes of dental pain reported in this study should be investigated further although sequelae of caries are the most likely reason for dental pain. This is so since 99% of the students investigated were without treatment experience in terms of tooth fillings provided by dental therapist, dentist or traditional healers. Dental pain estimates are recognized indicators of the oral health status as well as a measure of quality of life [31]. The present finding indicates that dental pain in primary schoolchildren could be avoided and thus their quality of life improved by strengthening preventive and therapeutic dental services in sparsely populated and remote areas of Tanzania.

Compared to the prevalence rate of Child-OIDP reported in 10–14-yr- old primary school children in Dar es Salaam (28%) [9], a higher prevalence rate was observed in Kilwa students, amounting to 36%. Nevertheless, the prevalence of OIDP observed in this study was lower than those reported among similar age groups in other cultures and also lower than those observed in East African adults [8]. Consistent with previous findings, the Child-OIDP index exhibited marked floor effect, amounting to 64%. Nevertheless, this inventory exhibited sufficient discriminative properties suggesting that it is suitable for detecting group differences in cross-sectional studies. The higher prevalence rate of oral impacts seen in urban students compared to their rural counterparts is in line with rural residents presenting a healthier profile in terms of self-reported pain and oral problems, although the level of parental education and family wealth was most favourable among urban residents (Table 1). Thus, Kilwa students from urban areas and of higher socio-economic status presented with higher prevalence of OIDP than did their rural- and lower socio-economic status counterparts. Socio-economic disparities in OHRQoL of younger age groups have been reported previously, however with low-income children having severe oral disease being those experiencing the poorest OHRQoL scores [32]. Eating and cleaning were the most frequently reported impairments in urban as well as rural areas, a finding that is consistent with those of other populations using the adult-and child versions of the OIDP instrument [33-36].

Consistent with pervious studies and irrespective of socio-economic position and dental caries experience, students reporting dental pain and oral problems during the last 3 months were more likely than their counterparts without such problems to present with impaired OIDP across the 8 impacts investigated [34-36]. As shown in table S2; additional file 2, dental pain was most strongly related to problems sleeping and difficulty to perform schoolwork and least strongly related to problems speaking-, smiling- and emotional stability. Thus, in Kilwa students, toothache seems to have more serious consequences for social- than for the functional and psychological performances. Contrary, reported oral problems were most strongly related to problem eating and cleaning and more weakly associated with other impairments. Obviously, the characteristics of symptoms (type, frequency and severity) that an individual experience would have varying consequences on different aspects of daily performances. As discussed by Locker [31], the psychosocial impacts of oral disorders tend to vary from individual to individual even though the severity of their clinical condition remains the same. Accordingly, Wong et al [37] studying the association between toothache and oral impacts in a sample of Hong Kong adults found toothache to be a stronger predictor of sleep- than of eating disturbances.

Understanding dental need perceptions is important for the effective planning and implementation of oral health care services. Consistent with theory and empirical findings, impaired OHRQoL was positively associated with perceived need for dental care in Kilwa students, indicating that a full understanding of young people's need for dental care cannot be captured by clinical indicators alone. These findings are consistent with previous reports, suggesting that self-evaluations of oral health status rather than disease presence per se are the primary determinants of perceived dental treatment needs [13,38,39]. Consistent with results of previous studies in older age groups, the present findings suggest that normatively assessed and perceived need for dental care differs among Tanzanian primary schoolchildren [38]. The present results provide insight into what oral impacts guide Kilwa students' perceived need for dental care. As shown in Table 2, respondents who reported problem eating, problem cleaning and problem sleeping were those most likely to perceive a need for dental care. Jokovic and Locker [39] found problems associated with chewing and appearance to be the impacts most strongly associated with perceived dental treatment need in adult populations. Future studies should compare the performance of various OHRQoL inventories for children in relation to reported dental pain and perceived need for dental care. Not everybody who perceived oral impacts reported need for dental treatment the latter being related to factors that predispose and enable individuals to express their needs. Thus, the least common discrepancy observed- in terms of reporting no treatment need whilst having impacts were most frequent in urban areas and among children in the less poor wealth categories. This indicates a social gradient in impairment coping- or impairment reducing behaviours, suggesting that urban children possess better ability to cope with adversity including impaired OHRQoL as compared to their rural counterparts. Hastie et al [40] suggested that besides seeking professional treatment, an individual can choose other pain and impairment coping strategies such as self-care, seeking of social support and spiritual/religious coping.

## Conclusion

Substantial proportions of students suffered from untreated dental caries, oral impacts on daily performances and perceived need for dental care. Dental pain and reported oral problems varied systematically with OIDP across the eight impacts considered. Eating- and tooth cleaning problems discriminated between subjects who perceived need for dental treatment and those who did not.

## Competing interests

The authors declare that they have no competing interests.

## Authors' contributions

KOM: Principal investigator, conceived of the study, designed the study, collected data, statistical analysis and manuscript writing. ANÅ: Main supervisor, designed study, statistical analysis and manuscript writing. JRM: Participated in design of study. DJ: Have commented on the paper and provided valuable guidance for manuscript write up/

## Supplementary Material

Additional file 1 **Table S1 – Subjects with oral impact on daily performance (each item) by socio-demographics, dental caries, dental pain and self reported dental problems**. Table showing subjects with oral impact on daily performance (each item) by socio-demographics, dental caries, dental pain and self reported dental problems. In this table **p < 0.001; and * p < 0.05. Click here for file

Additional file 2 **Table S2 – Oral impacts on daily performances by socio-demographics, dental caries, dental pain and dental problems**. Table showing the oral impacts on daily performances by socio-demographics, dental caries, dental pain and dental problems. In this table ^a ^Adjusted for age, gender, place of residence, parental education Click here for file
