# Supplementary material for: Dental pain, oral impacts and perceived need for dental treatment in Tanzanian school students: a cross-sectional study
Source: Health Qual Life Outcomes. 2009 Jul 30;7:73. doi: 10.1186/1477-7525-7-73 (PMC2726126; doi:10.1186/1477-7525-7-73)
Supplement: Additional file 1 — Table S1 – Subjects with oral impact on daily performance (each item) by socio-demographics, dental caries, dental pain and self reported dental problems. Table showing subjects with oral impact on daily performance (each item) by socio-demographics, dental caries, dental pain and self reported dental problems. In this table **p < 0.001; and * p < 0.05. [file 1477-7525-7-73-S1.doc]

Table S1; Subjects with oral impact on daily performance (each item) by socio-demographics, dental caries, dental pain and self reported dental problems

|  | Eating  % (n) | Speaking  % (n) | Cleaning  % (n) | Sleeping  % (n) | Smiling | Emotional | School work | Social contact |
| --- | --- | --- | --- | --- | --- | --- | --- | --- |
| Sex: |  |  |  |  | % (n) | % (n) | % (n) | % (n) |
| Male | 19.7 (171) | 12.3 (107) | 21.3 (185) | 13.3 (115) | 11.8 (102) | 14.0 (121) | 11.9 (103) | 12.3 (107) |
| Female | 18.8 (165) | 12.2 (107) | 17.7 (155) | 15.8 (139) | 11.0 (97) | 12.0 (105) | 12.0 (105) | 12.2 (107) |
| Age: |  |  |  |  |  |  |  |  |
| 10 – 14 years | 18.7 (221) | 11.9 (141) | 19.2 227) | 14.7 (174) | 11.2(133) | 12.8 (151) | 11.7 (139) | 12.4 (147) |
| 15 – 19 years | 20.5 (115) | 13.0 (73) | 20.1 (103) | 14.3 (80) | 11.8 (66) | 13.4(75) | 12.3 (69) | 11.9 (67) |
| Residence: |  |  |  |  |  |  |  |  |
| Urban | 22.8 (191)** | 14.2 (119)* | 22.8 (191)** | 17.2 (144)** | 12.5 (105) | 15.7 (131)** | 13.7 (115)* | 12.9 (108) |
| Rural | 16.0 (145) | 10.5 (95) | 16.4 (149) | 12.1 (110) | 10.4 (94) | 10.5 (95) | 10.2 (93) | 11.7 (106) |
| Mother’s education: |  |  |  |  |  |  |  |  |
| Low | 19.7 (154) | 11.8 (92) | 19.6 (153) | 16.0 (125) | 12.8 (100) | 14.4 (112) | 12.2 (95) | 11.9 (93) |
| High | 18.9 (182) | 12.6 (122) | 19.4 (187) | 13.4 (129) | 10.3 (99) | 11.8 (114) | 11.7 (113) | 12.5 (121) |
| Father’s education: |  |  |  |  |  |  |  |  |
| Low | 19.1 (139) | 11.7 (85) | 18.5 (134) | 14.9 (108) | 10.9 (79) | 12.4 (90) | 12.8 (93) | 11.8 (86) |
| High | 19.3 (197) | 12.7 (129) | 20.2 (206) | 14.3 (146) | 11.8 (120) | 13.3 (136) | 11.3 (115) | 12.6 (128) |
| Family wealth index: |  |  |  |  |  |  |  |  |
| 1^st^ quartile (Poorest) | 18.9 (87) | 13.2 (61) | 17.1 (79) | 14.8 (68) | 12.4 (57) | 11.9 (55) | 12.4 (57) | 10.2 (47) |
| 2^nd^ quartile | 15.9 (124) | 9.6 (75) | 16.3 (127) | 12.7 (99) | 10.5 (82) | 11.5 (90) | 10.5 (82) | 11.7 (91) |
| 3^rd^ quartile | 30.4 (21)** | 23.2 (16) | 29.0 (20)** | 20.3 (14) | 11.6 (8) | 15.9 (11) | 14.5 (10) | 15.9 (11) |
| 4^th^ quartile (Least poor) | 23.4 (104) | 14.3 (62) | 26.2 (114) | 16.8 (73) | 12.0 (52) | 16.1 (70) | 13.6 (59) | 14.9 (65) |
|  |  |  |  |  |  |  |  |  |
| Dental caries: |  |  |  |  |  |  |  |  |
| Yes | 30.7 (103)** | 19.7 (66)** | 26.0 (87)** | 28.4 (95) | 15.5 (52)* | 17.3 (277)** | 19.7 (66)** | 18.5 (62)** |
| No | 16.5 (223) | 10.5 (148) | 17.9 (253) | 11.3 (159) | 10.4 (147) | 11.9 (168) | 10.1 (142) | 10.8 |
| Dental pain: |  |  |  |  |  |  |  |  |
| Yes | 40.4 (213)** | 26.0 (137)** | 36.8 (194)** | 32.6 (172)** | 21.3 (112)** | 25.0 (132)** | 25.8 (136)** | 24.5 (129)** |
| No | 10.1 (123) | 6.3 (77) | 12.0 (146) | 6.7 (82) | 7.1 (87) | 7.7 (94) | 5.9 (729 | 7.0 (85) |
| Reported oral problems |  |  |  |  |  |  |  |  |
| Yes | 31.7 (268)** | 18.8 (159)** | 31.6 (267)** | 21.6 (267)** | 17.4 (147)** | 20.2 (171)** | 17.8 (151)** | 18.9 (160)** |
| No | 7.6 (68) | 6.1 (55) | 8.1 (73) | 7.9 (71) | 5.8 (52) | 6.1 (55) | 6.3 | 6.0 (54) |

**p<0.001; * p<0.05
