# Supplementary material for: Dental pain, oral impacts and perceived need for dental treatment in Tanzanian school students: a cross-sectional study
Source: Health Qual Life Outcomes. 2009 Jul 30;7:73. doi: 10.1186/1477-7525-7-73 (PMC2726126; doi:10.1186/1477-7525-7-73)
Supplement: Additional file 2 — Table S2 – Oral impacts on daily performances by socio-demographics, dental caries, dental pain and dental problems. Table showing the oral impacts on daily performances by socio-demographics, dental caries, dental pain and dental problems. In this table a Adjusted for age, gender, place of residence, parental education [file 1477-7525-7-73-S2.doc]

Table S2; Oral impacts on daily performances by socio-demographics, dental caries, dental pain and dental problems

|  | ^a^ Eating | ^a^ Speaking | ^a^ Cleaning | ^a^ Sleeping | ^a^ Smiling | ^a^ Emotion | ^a^ School work | ^a^Social contact |
| --- | --- | --- | --- | --- | --- | --- | --- | --- |
| Family wealth index | OR  (95% CI) | OR  (95% CI) | OR  (95% CI) | OR  (95% CI) | OR (95% CI) | OR (95% CI) | OR (95% CI) | OR (95% CI) |
| 1^st^ quartile (poorest) | 1 | 1 | 1 | 1 | 1 | 1 | 1 | 1 |
| 2^nd^ poor quartile | 0.6 (0.4-0.8) | 0.5 (0.3-0.8) | 0.7 (0.5-1.1) | 0.7 (0.4-1.0) | 0.7 (0.4-1.0) | 0.8 (0.5-1.2) | 0.7 (0.4-1.0) | 0.9 (0.6-1.4) |
| 3^rd^ poor quartile | 1.3 (0.7-2.4) | 1.5 (0.7-2.9) | 1.5 (0.5-1.0) | 1.0 (0.4-2.0) | 0.6 (0.3-1.5) | 1.0 (0.4-2.1) | 0.7 (0.3-1.7) | 1.3 (0.5-2.6) |
| 4^th^ quartile (least poor) | 0.8 (0.5-1.2) | 0.7 (0.4-1.1) | 1.1 (0.7-1.6) | 0.7 (0.5-1.1) | 0.6 (0.4-1.0) | 0.9 (0.6-1.4) | 0.7 (0.4-1.1) | 1.1 (0.7-1.7) |
| Dental caries :NO | 1 | 1 | 1 | 1 | 1 | 1 | 1 | 1 |
| Yes | 1.5 (1.1-2.0) | 1.4 (0.9-1.9) | 1.2 (0.8-1.5) | 2.2 (1.5-2.9) | 1.1 (0.7-1.6) | 1.1 (0.71.5) | 1.5 (1.0-2.0) | 1.3 (0.9-1.8) |
| Pain: No | 1 | 1 | 1 | 1 | 1 | 1 | 1 | 1 |
| Yes | 3.8 (2.9-5.1) | 3.8 (2.7-5.2) | 2.8 (2.1-3.6) | 4.7 (3.4-6.5) | 2.5 (1.8-3.6) | 2.6 (2.0-3.8) | 4.0 (2.9-5.7) | 3.1 (2.2-4.2) |
| Dental problem: No | 1 | 1 | 1 | 1 | 1 | 1 | 1 | 1 |
| Yes | 3.8 (2.8-5.2) | 2.3 (1.6-3.3) | 3.6 (2.7-4.9) | 1.9 (1.3-2.6) | 2.5 (1.8-3.7) | 2.6 (1.9-3.7) | 2.0 (1.4-2.9) | 2.5 (1.7-3.5) |
| **Nagelkerke’s R^2^** | **0.259** | **0.169** | **0.203** | **0.220** | **0.114** | **0.141** | **0.161** | **0.135** |

^a^ Adjusted for age, gender, place of residence, parental education
